# Influence of Point Mutations on PR65 Conformational Adaptability: Insights from Nanoaperture Optical Tweezer Experiments and Molecular Simulations

**DOI:** 10.21203/rs.3.rs-3599809/v1

**Published:** 2023-11-16

**Authors:** Ivet Bahar, Anupam Banerjee, Samuel Mathew, Mohsin Naqvi, Sema Yilmaz, Maria Zachoropoulou, Pemra Doruker, Janet Kumita, Shang-Hua Yang, Mert Gur, Laura Itzhaki, Reuven Gordon

**Affiliations:** Stony Brook University; Stony Brook University; University of Victoria; University of Cambridge; Istanbul Technical University; University of Cambridge; University of Pittsburgh; University of Cambridge; National Tsing Hua University; University of Pittsburgh; University of Cambridge; University of Victoria

**Keywords:** phosphatase, PP2A, PR65, point mutations, protein dynamics, optical tweezers, molecular dynamics simulations, hinge sites

## Abstract

PR65 is the HEAT-repeat scaffold subunit of the heterotrimeric protein phosphatase 2A (PP2A) and an archetypal tandem-repeat protein, forming a spring-like architecture. PR65 conformational mechanics play a crucial role in PP2A function by opening/closing the substrate-binding/catalysis interface. Using *in-silico* saturation mutagenesis we identified “hinge” residues of PR65, whose substitutions are predicted to restrict its conformational adaptability and thereby disrupt PP2A function. Molecular simulations revealed that a subset of hinge mutations stabilized the extended/open conformation, whereas another had the opposite effect. By trapping in nanoaperture optical tweezer, we characterized PR65 motion and showed that the former mutants exhibited higher corner frequencies and lower translational scattering, indicating a shift towards extended conformations, whereas the latter showed the opposite behavior. Thus, experiments confirm the conformations predicted computationally. The study highlights the utility of nanoaperture-based tweezers for exploring structure and dynamics, and the power of integrating this single-molecule method with *in silico* approaches.

## Introduction

Maintaining cellular signaling and homeostasis is crucial for the proper functioning of living organisms, and dysregulation of these processes can result in the development of many diseases. A complex interplay between kinases and phosphatases contributes to signaling events and cellular homeostasis^1^. Abnormal activation of kinases and inactivation of phosphatases can lead to pathological hyperphosphorylation, a key factor in the development of numerous diseases, including cancer and neurodegenerative disorders^2, 3^. Much attention has been given to kinase inhibitors for the treatment of these diseases. Phosphatases, on the other hand, have been much less studied as drug targets^4–9^ mainly due to the lack of druggable pockets near their active sites^10, 11^.

**Figure 1:**
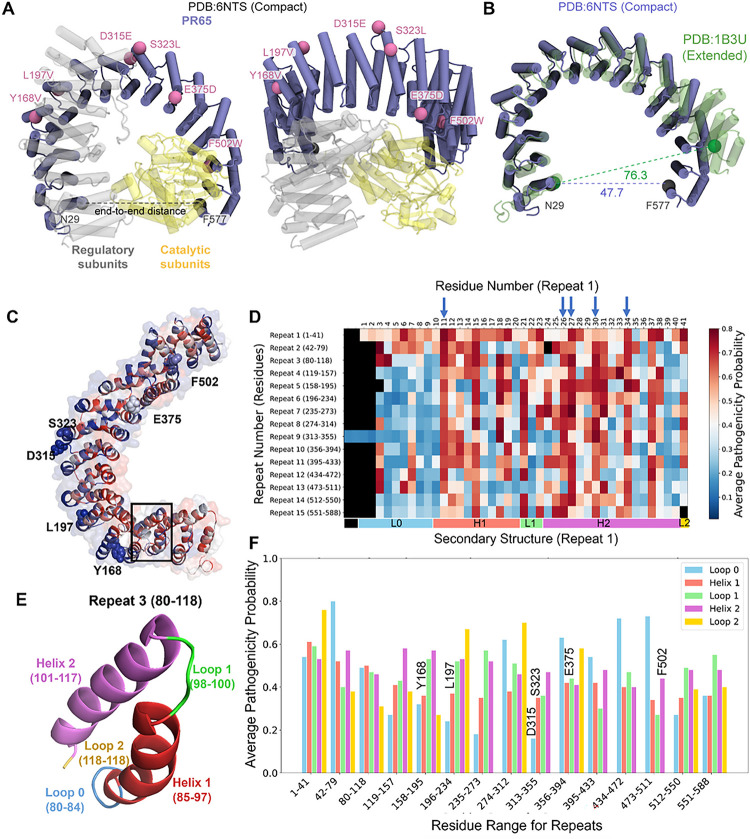
PP2A heterotrimer, conformational adaptability and potential pathogenicity of its scaffolding subunit PR65. **(A)** Two different views of the PP2A trimer (Protein Data Bank (PDB): 6NTS^3^) with PR65 shown in *navy/mauve*, and the catalytic and regulatory subunits in *yellow* and *gray*, respectively. The locations of six residues mutated in the present study are indicated by beads. (**B**) Compact (solid) and extended (*green*, transparent; PDB: 1B3U^35^) conformations of PR65 in the trimer and in isolation, respectively. N29 and F577 are used as references to define the end-to-end extension^36^. (**C**) PR65 color-coded by average pathogenicity of residues as predicted by Rhapsody. The color ranges from *blue* (lowest probability of being deleterious; 0.12 here) to *red* (highest probability, 0.80). Note that mutations at the inner portions of the repeats generally tend to have more deleterious effects compared to the outer regions. (**D**) Heat map showing the average pathogenicity of residues (*abscissa*) within each of HEAT repeat (*ordinate*). Non-existent residue positions are shown in *black*. (**E**) The loop0-helix1-loop1-helix2-loop2 motif of HEAT repeats, illustrated here for Repeat 3. Helix 1 is outer and helix 2 is inner. (**F**) Average pathogenicity probability for the helices and loops of each HEAT repeat. Consistent with panel **A**, helix 2 tends to exhibit higher sensitivity to mutations than helix 1; whereas the inter-repeat loops 0 and 2 exhibit higher pathogenicity than the intra-repeat loop 1. The color-coded secondary structure corresponding to the first repeat is shown along the abscissa (*bottom*) of panel **B**. Note that not all repeats have the same helix and loop lengths.

One major class of phosphatases playing a central role in maintaining cellular homeostasis is the family of serine/threonine protein phosphatases 2A (PP2A)^12–14^. PP2A is frequently dysregulated in human diseases, making it an attractive target for therapeutic interventions^15^. It is a heterotrimer, composed of a scaffold (A) subunit, known as PR65, a catalytic (C) subunit, and one of many regulatory (B) subunits (Figure 1A). The A and C subunits form the core of PP2A.The specificity of PP2A is controlled by the choice of the regulatory subunit that binds the AC core, with over 40 different B subunits each determining the specific substrate bound to PP2A^3^. The diverse array of B subunits allows PP2A to exert control over a majority of cellular signaling pathways.

PR65 serves as a structural scaffold that provides a platform for the assembly of the heterotrimer^16^. Among the three PP2A subunits, PR65 experiences the highest frequency of mutations, which have been implicated in altering PP2A activity^17–19^. Understanding the impact of PR65 point mutations on PP2A structure and function is essential to unraveling the mechanisms underlying various diseases and developing targeted therapeutic strategies^20^. PR65 is a tandem repeat (TR) protein consisting of 15 HEAT repeats, each comprising ~ 40-residue antiparallel helices. These repeats stack in a one-dimensional fashion, forming an elongated, horseshoe-like superhelical structure composed of outer and inner helices layers. Many TR proteins act as hubs in multiprotein complexes, whereby their conformational fluctuations facilitate the function of the assembly^21–23^. In the case of PR65, its mechanics play a crucial role in regulating PP2A function; the collective motions of PP2A mediated by PR65, open and close the enzyme’s substrate binding/catalysis interface. Maintaining the flexibility of PR65 to sample alternative conformers is crucial, with the closed state being active and facilitating the formation of the PP2A complex with catalytic and regulatory subunits^24, 25^. Although the effects of PR65 mutations on folding and binding energetics have been studied^17–20^, a systematic investigation of the modulation of PR65’s conformational state, flexibility, and adaptability by point mutations is lacking. In this study, we aim to fill this gap and explore the effects of point mutations on the structure and dynamics of PR65. We focus on a unique aspect of PR65 structural mechanics—the so-called ‘hinge’ sites that coordinate the global dynamics of the entire complex. Hinge regions usually play a key role in mediating the conformational mechanics of the proteins and enabling conformational changes that underlie functional transitions^26^.

By specifically targeting mutations at the hinge sites, we aim to investigate how subtle alterations in these regions influence the conformational space accessible to PR65. Our approach is to examine the changes in the conformations and dynamics of PR65 induced by introducing point mutations at those sites. We hypothesize that certain mutations may restrict the conformational adaptability of PR65, potentially impacting the formation of the PP2A complex and its activity. To assess the effect of point mutations, we integrated molecular dynamics (MD) simulations and elastic network model-based analysis^27^ and experimentally tested the findings using nanoaperture optical tweezer measurements^28^ .

An outstanding challenge in biology is how to visualize protein structure at the single molecule level and on functionally relevant timescales without the use of labels, immobilization, or tethering, which can result in artifacts that perturb the system we are trying to observe^29^. Here we present nanoaperture optical tweezers. Optical tweezers have emerged as a powerful tool for probing the biophysics of proteins at the molecular level. The present study demonstrates the utility of the enhanced field confinement and sensitivity provided by nanoaperture-based tweezers for studying the structure and dynamics of single proteins. Unlike conventional optical tweezer techniques, this approach allows for characterization of individual unmodified proteins in solution for extended durations and without the need for tethers or labels^30, 31^.

Using site-directed saturation mutagenesis *in-silico*^32, 33^, six PR65 mutants (Y168V, L197V, D315E, S323L, E375D, and F502W) because of their colocalization with the global hinge sites. Extensive MD simulations revealed that S323L, E375D, and F502W favored the adoption of relatively more extended conformations compared to the wild type (WT), while the conformational flexibility enabling the fluctuations between open and closed conformers was relatively suppressed. The Y168V and D315E mutants, in contrast, favored relatively more compact conformations with mixed effects on conformational dynamics. The F502W mutation helped to regulate the transitions between the compact and extended form in a more favorable way than the WT PR65. D315E, on the other hand, significantly perturbed the overall stability and dynamics, interfering with the ability of PR65 to adapt to conformational changes required for function. Concurrently, by trapping the mutants in the optical tweezer setup, we measured their motion and observed that the S323L, E375D, and F502W mutants shifted towards extended conformations, exhibited by higher corner frequencies and lower root-mean-square-deviations (RMSD) in their amplitudes of motions compared to the WT PR65. Since elongation typically leads to a higher polarizability^34^ and therefore larger optical tweezer stiffness, these findings appear to independently confirm the conformational variability for all six mutants.

Overall, our study represents an integration of results from *in-silico* saturation mutagenesis, MD simulations, and optical tweezers experiments to characterize the conformational mechanics of PR65 and its mutants. The combination of these complementary approaches provides insights into the modulation of PR65 conformational flexibility and adaptability, laying the foundation for further investigations and potential therapeutic interventions. It also offers an integrated protocol for exploring the structural and dynamic consequences of mutations, generalizable to other systems and highlights the utility of optical tweezers in molecular biophysics applications.

## Results

### In-silico Saturation Mutagenesis of PR65.

The *in-silico* saturation mutagenesis study of PR65 was performed using a recently introduced structure- and dynamics-based machine learning methodology, implemented in the online accessible tool *Rhapsody*^32, 37^ (Figure S1). The approach allows for assessing the impact, neutral or pathogenic, of any substitution at any residue along the protein based on sequence (conservation and co-evolution), structure (accessible surface area), and dynamics (equilibrium fluctuations, allosteric couplings, and mechanical behavior) of the protein. In parallel, we estimated the change in folding free energies associated with point mutations using ProTSPoM^33^ (Figure S2A). ProTSPoM uses residue physicochemical and energetic properties in the folded state, environmental compatibility, and evolutionary information to predict the change in Gibbs free energy (DDG) of folding associated with point mutations.

Figure 1C–F presents the results for the apo structure of PR65. The diagram in panel C is color-coded by average pathogenicity score for each residue *I*, i.e., the probability of having a deleterious/pathogenic effect upon mutating the *i*^*th*^ residue, averaged over all 19 amino acid substitutions at that position. The effects of the individual substitutions are described by the elements of the *i*^*th*^ column in the saturation mutagenesis heat map (see Figure S1 for a PR65 segment). The scores vary from 0 (neutral, *blue*) to 1 (strongly pathogenic, *red*), with a cutoff of 0.65 determining the decision between neutral and deleterious. Using the residue ranges defined earlier^36^, we evaluated the pathogenicity scores of the residues within each of the 15 HEAT repeats. The results are presented in Figure 1D, organized by repeat number (*ordinate*) and corresponding residue positions (*abscissa*). The heat map shows that the counterparts of the repeat 1 residues P11 (in helix 1), L26, R27, S30, and L34 (in helix 2) in all repeats consistently exhibit high pathogenicity probabilities. These residues are indicated by *blue* arrows along the abscissa. Their resistance to tolerate mutations is consistent with their high degree of sequence conservation at those positions, usually occupied by hydrophobic residues (leucine, valine, and isoleucine) or by arginine. See the counterpart of this heat map corresponding to change in free energy of folding, ΔΔG, in Figure S2B.

Closer examination of HEAT repeat structural elements (loop 0, helix 1, loop 1, helix 2 and loop 2; Figure 1E) revealed the distinctive behavior of inter- and intra-repeat elements. In Figure 1F we present the average pathogenicity for these structural elements. The corresponding residue ranges are listed in Table S1^36^. Notably, the loops 0 and 2 linking successive repeats generally exhibit relatively high pathogenicity if mutated, suggesting the high sensitivity of PR65 if not inability to tolerate mutations at inter-repeat regions. See the peaks in Figure 1F between repeats 1–2, and those at the loop 0 or 2 between repeats 6–7, 8–9, 10–11, 11–12, and 12–13. The latter two represent kinks of single residues (G434 and G473, respectively), rather than loops, that presumably play a critical role. The counterpart of this analysis for ΔΔG is presented in Figure S3, which also draws attention to the critical role of S119 between repeats 3–4.

This analysis therefore identified the inter-repeat loop residues to generally play a critical role in ensuring the overall stability and/or functional flexibility of PR65. Closer analysis also identified specific mutations at the regulatory and catalytic subunit interfaces of PR65 that would induce the strongest destabilization and pathogenicity. Table 1 lists these mutations, Figure S4 displays their location in the PP2A structure. We note again the propensity of helix 2 residues among these critical sites.

### Selection of Hinge Site Mutations.

**Figure 2:**
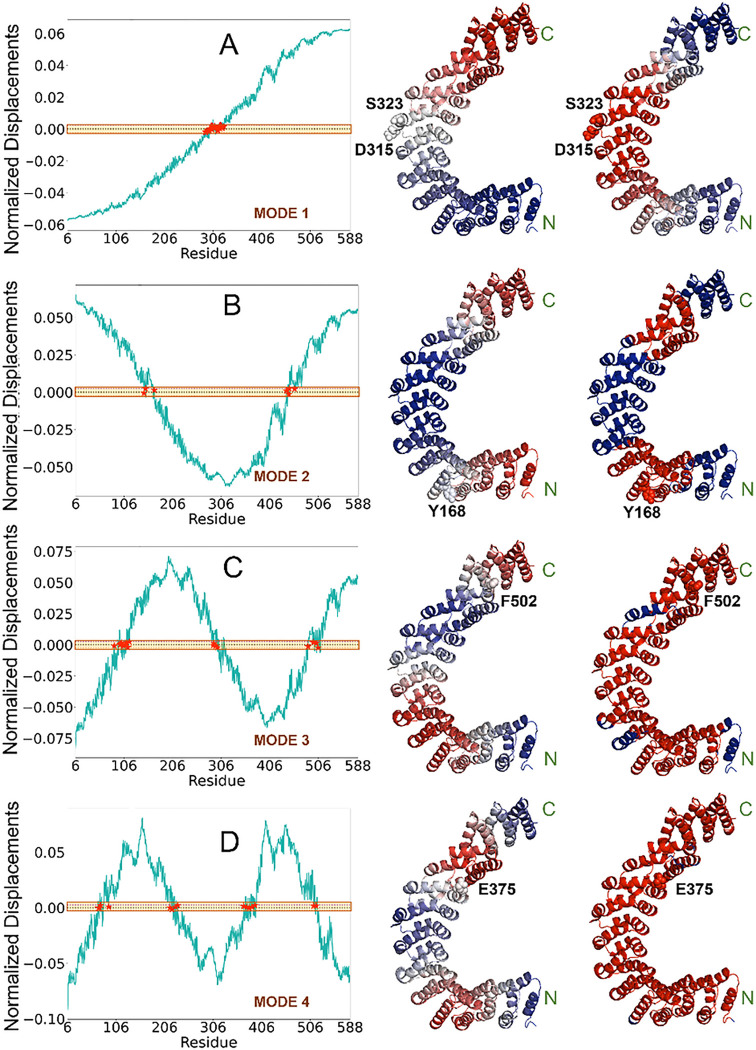
Hinge residues facilitate conformational transformation of PR65. **(A) Results for mode 1.** The left curve shows the normalized displacements of PR65 residues along GNM mode 1. The region within the band 0±0.0025 (shown in *yellow shade*) indicates the crossover between positive and negative direction motions along the mode axis, and the residues lying in this region (shown by *red* stars) act as global hinge sites. The middle ribbon diagram is color-coded according to the displacement along this mode, varying from *blue (negative)* to *red (positive)*. Hinge sites are at the crossover between blue and red regions. The *right* diagram is color-coded by the size of the displacements from *red* (minimum displacement) to *blue* (maximum). Hinge sites are colored *red*, as they are subject to minimal displacements if any. Selected hinge site residues are labeled in each case. The corresponding mutants (listed in Table S2) were found to be expressed and soluble in significant amounts, enabling further investigation of their impact on PR65. The same results are presented for GNM modes 2–4 in the respective panels **(B)**-**(D)**.

Our goal in this study was to explore the possibility of altering the conformational state and flexibility, and thereby function, of PR65 without completely destabilizing the scaffold or abolishing its function. Toward this goal, we turned our attention at residues predicted to play a key mechanical role as hinges/anchors during cooperative movements (global modes of motion) of PR65. We focused on 21 residues predicted by the Gaussian network model (GNM)^38, 39^ to participate in hinge regions modulating the softest six modes (Table S2) and explored how substitutions at those sites could alter the PR65 structure and dynamics. Figure 2A–D shows the shapes of GNM modes 1–4 (*left*) and corresponding diagrams color-coded by the direction (*middle*) and size (*right*) of residue displacements along those modes.

Based on the pathogenicity scores and ΔΔG values predicted for selected point mutations at those hinge sites (Table S2), we categorize the hinge site mutations into two broad groups, (a) pathogenic and (b) non-pathogenic depending on their pathogenicity scores. The former group also entails an increase in ΔG, indicating that these mutations would be destabilizing. The latter group, on the other hand, which is of interest as potential mutations that can alter the function while retaining the fold, is divided into three subgroups based on their DDG values: (b1) not destabilizing (negative ΔΔG), (b2) mildly destabilizing (ΔΔG < 1.25 kcal/mol), and (b3) highly destabilizing ΔΔG ≥ 1.25 kcal/mol, as mentioned in Table S2.

### Experimental assessment of the thermodynamic stability of mutants.

We previously used *E. coli* to express PR65 WT and mutants for folding studies^20^. To test the thermodynamic stabilities of the mutants in Table S2, we first examined the mutations in group (a), which were predicted to adversely affect the protein or function (pathogenicity score > 0.65). As seen in Table S2, the group comprises of 12 mutations. Most of them are also predicted to be highly destabilizing (DDG ≥ 1.25 kcal/mol). We performed small-scale protein expression tests in *E. coli* on them and the results showed that 11 of these mutants had no expression or were insoluble, indicative of instability and thus consistent with the predicted impact of these mutations from the computational analysis. On the other hand, out of the six mutations predicted to be stabilizing and non-pathogenic (subgroup b1), three - D315E, S323L, E375D - were expressed in good quantity. One mildly destabilizing and non-pathogenic mutation (subgroup b2), F502W, was also successfully expressed. Notably, two subgroup b3 mutations Y186V, and L197V (predicted to be neutral by Rhapsody but destabilizing by ProTSPoM), were also expressed in good yield.

We next performed large-scale expression of these six mutants and used thermal unfolding to qualitatively assess their thermodynamic stabilities as measured by melting temperature (the temperature at which the protein is 50% unfolded) (Table S3). All mutants had melting temperatures within 1 °C of the WT value (51.3 °C), indicating that the mutations had only very small effects on stability. Therefore, experiments were in general in accordance with predicted potential pathogenicity; and they were also consistent with the predicted mild-to-none effects of mutations on fold stability except for the b3 mutations. We therefore moved on to examine the impact of these hinge site point mutations on the structure, dynamics, and potentially function, of PR65 by molecular dynamics (MD) simulation and optical tweezer experiments.

### MD simulations indicate that the mutants S323L, E375D, and F502W preferentially sample extended conformations.

**Figure 3:**
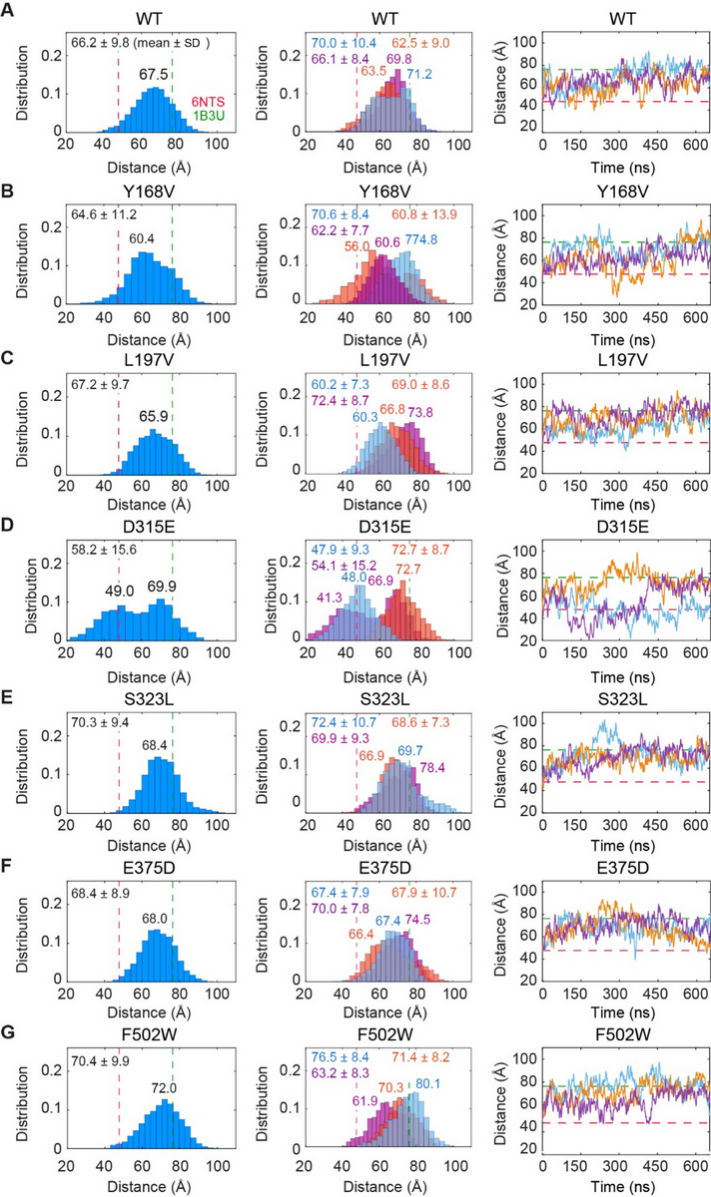
The mutants S323L, E375D and particularly F502W sample more extended conformations than WT PR65 (**A**) End-to-end distances distributions and time evolution for WT PR65. *Left* and *middle* panels show the distribution of end-to-end distances for the combined MD trajectory (*blue*) and each set of simulations separately (*light blue, magenta*, and *orange*), respectively. The mean ± standard deviation is indicated in each histogram. Location of peaks are indicated on the distributions. *Dashed lines*represent the end-to-end distances observed in the crystallographically resolved compact (PDB: 6NTS) and extended (PDB: 1B3U) structures of PR65. The *right* panel shows the time evolution of the end-to-end distance. (**B-G**) Same as **A** for the indicated mutants.

Simulations were initiated from the compact form of PR65, taken from the heterotrimeric PP2A^3^. The distributions of the end-to-end distances, defined as the distance between the C^α^ atoms of N29 and F577 (as in earlier work^36^), are presented in Figure 3A and B–G (six mutants). In each case, the average histogram deduced from triplicate runs is shown in the *left* panel, and the individual histograms from each of the three runs are shown in the *middle*. The mean values and standard deviations (SD) are written in each case, and their averages over triplicate runs are reported in Table 2 *columns* 3 and 4. The panels on the *right* of Figure 3 show the time evolution of the end-to-end distance for each run.

Consistent with our prior study^16^, WT PR65 (Fig 3A) samples a broad range of end-to-end distances, from 36.3 Å to 95.8 Å, including those resolved for the compact (47.7 Å; see Figure 1B) and extended structures (76.3 Å), with a mean value of 66.2 Å. All three independent runs consistently exhibited similar distributions.

The triplicate runs conducted for each of the mutants S323L, E375D, and F502W also showed overlapping distributions of end-to-end distances despite small shifts (*middle* histograms in Figure 3E–G). However, the main difference from the WT PR65 was the shifts in the end-to-end distances towards more extended states. The corresponding mean end-to-end distances (70.3, 68.4 and 70.4 Å, respectively; cumulative histograms on the *left*) are larger than that of the WT. Thereby, these mutations favor more extended conformations in comparison to the WT PR65.

In contrast, the mutants Y168V and L197V (Figure 3B–D) were observed to sample end-to-end distances comparable to that of the WT PR65, if not more compact forms. D315E was able to sample much more compact conformations (as low as 17.5Å) than WT and gave the lowest mean end-to-end distances. For Y168V, L197V, and D315E, the main effect of mutations seems to compromise the ability of the structure to uniformly sample the conformational space; instead, the individual runs tend to gravitate/drift towards different forms, as evidenced by the histograms (*middle diagrams*) that show only a partial overlap. This effect was particularly pronounced in the mutant D315E, where two of the runs sampled rather compact forms with new peaks appearing at end-to-end distance of 41.3 and 48.0 Å -; whereas the third run sampled an extended form (mean value of 72.7 Å) with no transition to the compact form (Figure 3D).

### The interface between repeats 12 and 13 significantly contributes to the opening and closing of PR65.

**Figure 4:**
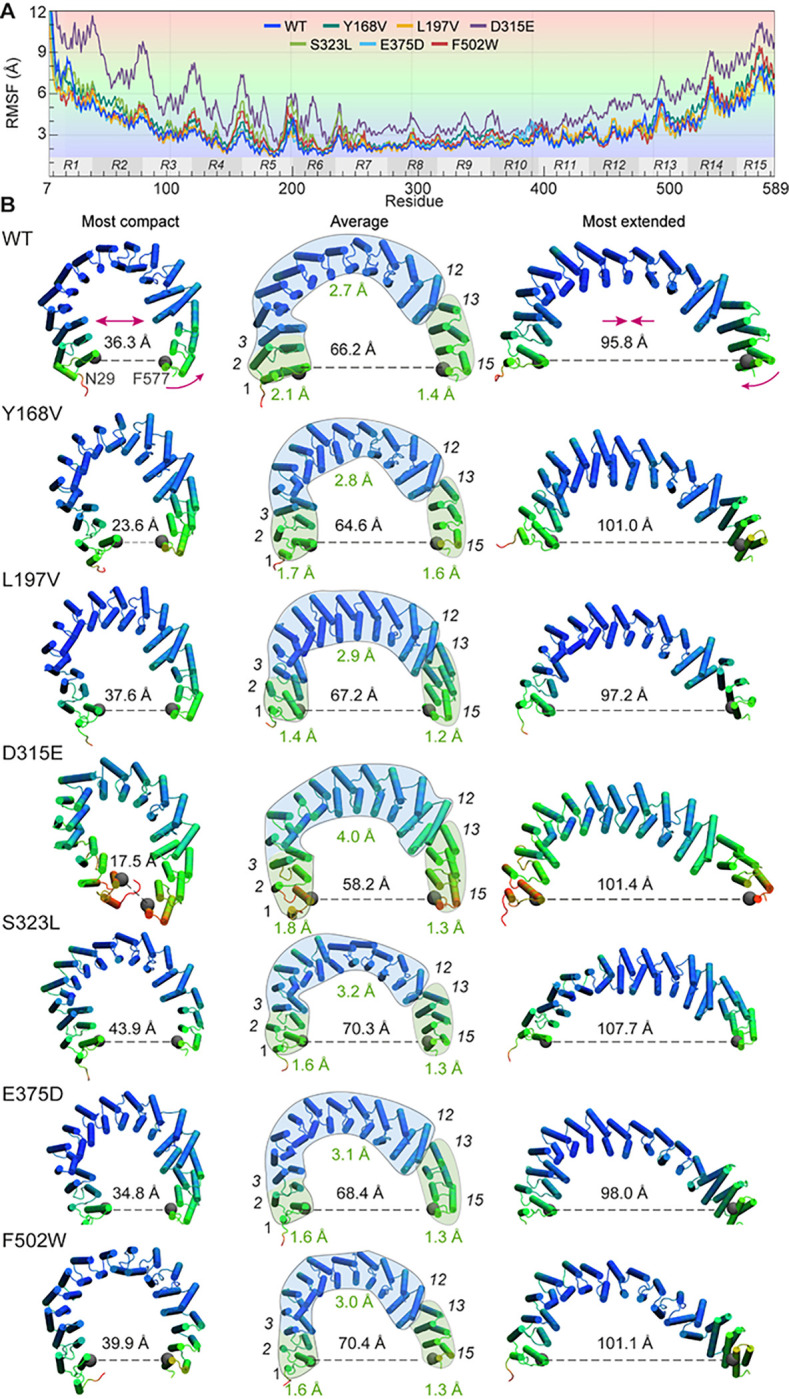
Residue mobilities observed for WT PR65 and its mutants. (**A**) RMSF profiles of PR65 residues observed in MD simulations for the WT PR65 and six mutants. (**B**) Conformations sampled during simulations. Each row illustrates the most compact (*left*), average (*middle*), and most extended (*right*) forms. The residues are colored in accordance with their RMSFs, as delineated in panel A. The end-to-end distances are shown with a dashed line. The mean internal RMSD corresponding to each section (C-terminal, middle and N-terminal; separated by the *green, blue*, and *green* shades in the middle diagrams) are annotated in green. Internal RMSDs are obtained by separately superposing the three different sections. The C-terminal section shows relatively small internal RMSDs, while it undergoes large displacements with respect to the remaining portions, enabled by a rigid-like reorientation at the interface between repeats 12 and 13.

Figure 4 displays the root-mean-square-fluctuations (RMSF) profile of residues (average size of fluctuations observed in triplicate trajectories). The regular patterns of the repeat units can be distinguished. Panel B displays the mutants colored by their RMSFs, in line with the shades in panel A. Examination of the RMSFs shows that the structure can be divided into three substructures: a middle section comprised of repeats 3–12 that shows small displacements (in *blue*), flanked by two segments (N-terminal repeats 1–2 and C-terminal repeats 13–15) that move significantly in space (in *green*).

Further examination of these individual sections shows that their spatial displacements do not necessarily reflect their conformational flexibilities. For example, even though the middle section is subject to minimal motions, it undergoes substantial *internal* rearrangements or deformations, as measured by the internal RMSDs (between 2.7 Å and 4.0 Å) evaluated for each mutant (by aligning with compact PR65 (PDB: 6NTS)). These rearrangements are presumably required to accommodate the local conformational fluctuations with minimal effects on the flanking regions. In contrast, the N- and C-termini that significantly move in space show much smaller internal RMSDs indicative of *en-bloc* movements of the repeats. In particular, the C-terminal section undergoes such *rigid* overall reorientation with respect to the middle section, enabled by hinge-bending at the interface between repeats 12 and 13. The internal RMSDs are confined in this case to 1.2–1.6 Å. These rigid-body movements of the C-terminal section, combined with the conformational rearrangements of the remaining structure, enable the opening/closing of PR65 that may sample compact and extended forms, as illustrated in Figure 4. Notably, F502W, located at the inner helix (helix 2) of repeat 13 near this hinge region, significantly alters PR65 equilibrium dynamics in favor of more extended conformations, underscoring the mechanical significance of this particular site.

### Compared to WT PR65, F502W exhibits an increased ability to transition between open and closed forms, whereas D315E exhibits a decreased ability.

To further investigate the effect of these point mutations on PR65 structural dynamics, we evaluated the correlation cosines between the global movements observed during the simulations and the deformation vector representing the experimentally observed difference between the compact and extended PR65 structures. The global movements sampled in MD simulations were characterized by principal component analysis (PCA)^40^. The PCA was performed using the triplicate MD trajectories (total of 1.962 μs) generated for WT PR65 and each of the six mutants. First, we aligned the conformations observed in MD with the compact PR65 structure to exclude rotational and translational motions. Thus, PCA yields 3*N*-6 internal motions. The first principal component (PC1) describes the most dominant mode of collective motion, which is also energetically favorable (*soft* mode), succeeded by PC2 and PC3.

**Figure 5:**
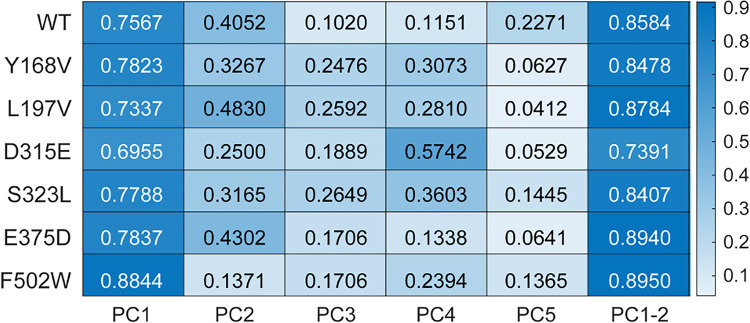
Correlation cosines between the PCs sampled in simulations and the deformation vector experimentally observed between the compact and extended forms of PR65. The correlation cosines between the five most globally significant PCs of the WT and mutants derived from the combined (triplicate) trajectories, and the deformation vector that delineates the transition from compact to extended PR65 structures. The x-axis refers to PC1-PC5, individually, and the last column show the cumulative effects of PC1–2. The correlation cosines are color-coded for clarity: *White* signifies no correlation, whereas *blue* indicates strong correlation.

Our goal was to assess whether the mutations impaired (or enhanced) the ability of PR65 to undergo its functionally required transitions between extended and compact forms. To this aim, we calculated the 3N-dimensional deformation vector pointing from the compact (PDB: 6NTS) to the extended (PDB: 1B3U) structures and examined if/how the PCs derived from MD simulations correlated with this structural change. As a quantitative measure of the ability of the PR65 mutants to effectuate these functional movements, we used the correlation cosine between the PCs (from simulations) and the deformation vector (from experiments)^16^. The heatmap in Figure 5 presents the results for all simulated systems. First, we note that the WT PR65’s PC1 yields a correlation cosine of 0.756 with the experimentally observed structural change, indicating this mode’s ability to predict 75.6% of the structural transition between the compact and extended PR65 structures. This is in accord with the previously reported intrinsic ability of the scaffold to accommodate, if not drive, these functional changes in structure. More important is to see to what extent the mutants exhibit similar abilities. In the cases of D315E and L197V mutants, PC1’s ability to describe the conformational transition between the compact and extended forms decreased to 69.55% and 73.37% respectively, indicating a small loss in the ability of the mutant to accommodate these changes in structure. In contrast, Y168V, S323L, and E375D, yielded values of 78.23%, 77.88%, and 78.37%, respectively, suggesting that the PR65 global dynamics is robust to those point mutations. Strikingly, the PC1 of F502W stood out from the others by yielding a correlation cosine of 0.8844 between PR65’s compact and extended forms, which is even higher than that of the WT. This result draws attention to the significance of F502 on PR65’s functional dynamics (and enhancing it when replaced by a tryptophan), which may have important ramifications for redesign or alteration of catalytic activity.

We further evaluated the cumulative correlation cosines with subset of 2 modes (PC1–2 in Figure 5), in accord with our previous study^41^. The cumulative correlation cosines for F502W PC1–2 reaches 0.895, which is higher than that of WT (0.858). D315E results in lower cumulative correlation cosines (0.739 in PC1–2) than the WT. Therefore, our findings suggest that the single point mutations F502W could impart alterations in protein dynamics that promote the ability of PR65 to undergo transitions between the compact and extended structures to accommodate trimeric assembly or diverse regulatory subunit binding. Conversely, D315E nudges the system towards a state that less favorably accommodates such transformations. These shifts could potentially impact the function of PR65.

### Nanoaperture Optical Tweezer based characterization of the PR65 protein and its variants.

**Figure 6:**
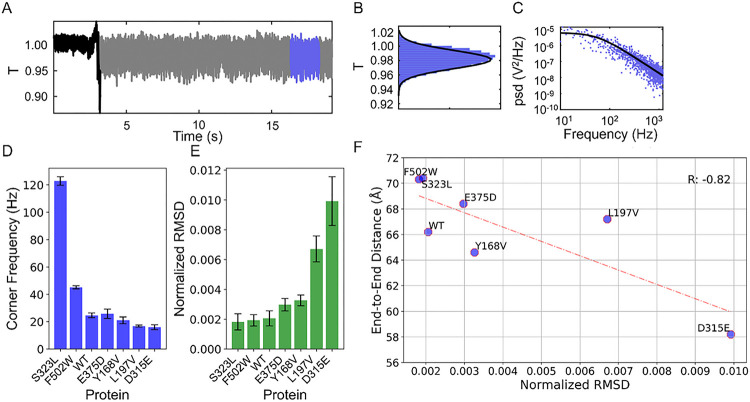
Tetherless aperture-based nanoplasmonic tweezers enable characterization of the structural dynamics of PR65 and single-site mutants. (**A**) PR65 undergoes a characteristic step as it enters the double nanohole trap, followed by an increase in noise from Brownian translational motion. (**B**) A histogram of this noise (transmission through the aperture, T, normalized to the pre-trap level) sampled after the trapping event. (**C**) The power spectral density from the detected transmission of PR65. (**D-E**) RMSD (**E**), and corner frequency (**D**) of the amplitude of the noise for WT PR65 and the six mutants. (**F**) The mean end-to-end distance observed across 1.962 μs of MD simulations for each of the seven investigated proteins (WT PR65 and the six mutants) report a Pearson correlation coefficient of −0.82 with the normalized RMSD. The standard deviation in end-to-end distances corresponding to MD simulations report a Pearson correlation coefficient of 0.83 with the normalized RMSD from experiments (Figure S5).

Optical tweezers have been used widely to probe the biophysics of proteins at the single molecule level^42–44^. By using enhanced field confinement and sensitivity, nanoaperture-based plasmonic tweezers have been adopted by several groups to study single proteins, protein complexes and their interactions without the need for (or potential impact from) tethers or labels^30, 31, 45–49^. Here we trap the protein using double nanoholes^50^ fabricated by a random colloidal lithography technique^28^. A 980 nm laser with a 1.3 NA 100× objective is focused on the aperture, with 22.5 mW of power incident on the aperture in a diffraction limited spot. The transmission through the aperture is monitored on an avalanche photodiode (using a 1.3 OD filter to prevent saturation). When trapped, the PR65 protein undergoes a characteristic step as it enters the trap, with an increase in the noise amplitude, as shown in Figure 6A.

Once in the trap, the Brownian motion of the particle results in increased “noise” in the photodiode signal (transmission through the aperture, T, normalized to the pre-trap level). A histogram of this noise sampled after the trapping event is given in Figure 6B. The stiffer the optical tweezer potential, the less motion that the particle undergoes^42^, so the amplitude of the noise is expected to be smaller (RMSD). The stiffness is proportional to polarizability of the particle, which is higher for longer particles, so less deviation is expected if the protein is extended by a point mutation. Another quantity that can be extracted from the detected transmission is the power spectral density shown in Figure 6C. This has a corner frequency proportional to the trap stiffness divided by the hydrodynamic drag on the protein^42^ and so it is expected to show the opposite trend of increasing as the particle lengths (opposite to the RMSD). These two quantities for the point mutations are compared with the WT in Figure 6D–E and Table 2.

As noted above, S323L, E375D, and F502W shifted towards the extended conformation, and these showed the highest corner frequencies among all studied mutants as well as the WT PR65 (Figure 6D). They also showed the lowest RMSD (Figure 6D), however, E375D was larger than the WT. We stress that this RMSD is the result of Brownian motion of the protein in the optical potential, and therefore reflect both overall tumbling and global internal motions, different from the internal motions seen in MD simulations which occur at a much faster timescale, even though the end-to-end fluctuations observed in MD and the extracted PCs reflect relatively slow events. Both of these findings separately confirm the *in-silico* prediction that the protein is extended in the presence of these three mutations S323L, E375D, and F502W. The mutants L197V, Y168V and D315E, on the other hand, showed the opposite trend in the experiments, and this confirms the *in-silico* predictions of a more compacted form (Figure 6F). Finally, we note that the most dramatic behavior departing from the WT (and other mutants) has been observed in the mutant D315E, which is also consistent with MD results where D315E is distinguished by the impact of the point mutations on its structure (Figure 4) and functional dynamics (Figure 5).

## Discussion

PP2A is a heterotrimeric Serine/Threonine protein phosphatase that plays a significant role in maintaining cellular processes and signaling pathways and whose dysregulation has been linked to multiple cancers, Alzheimer’s disease, and increased susceptibility to pathogen infections. In this study, we explored the effects of point mutations on the structure and dynamics of PR65, the scaffold subunit of the heterotrimeric PP2A complex. By targeting specific mutations at the hinge sites of PR65, we aimed to investigate how subtle alterations in these regions can influence the conformational space accessible to PR65 and potentially impact the assembly and function of the PP2A complex. Our *in-silico* analysis revealed that mutations at certain hinge sites, such as S323L, E375D and particularly F502W, stabilized relatively more extended conformations of PR65, whereas other mutations, e.g., Y168V and specifically D315E, favored a more compact form. In addition, mutations F502W and D315E affected the global dynamics of PR65, though they did so in contrasting manners: F502W enhanced the ability of PR65 to accommodate its structural transition between its compact and extended forms, whereas D315E had the opposite effect.

We leveraged the power of optical tweezers to analyze the changes in conformational flexibility of both the WT PR65 and its mutants at the single-molecule level. Unlike conventional optical tweezers that use labels and/or tethers, we used nanoplasmonic optical tweezers that allow for studying the unmodified protein at the single-molecule level. Comparison of the optical tweezer data obtained for PR65 mutants with those observed in the WT PR65, revealed trends consistent with the molecular simulations. The mutants S323L, E375D, and F502W showed the highest corner frequencies in the experimental measurements, indicating that these mutations resulted in higher trap stiffnesses and more extended conformations, and the opposite trend was observed for the other mutants.

This study illustrates how our recently developed *in-silico* saturation mutagenesis screen can identify critical residues where mutation is associated with decreased functionality while retaining their fold. The findings contribute to a better understanding of the changes in the structure and dynamics of PR65 as a function of amino acid substitutions, thus providing new insights for rational modification or redesign of its function. Importantly, the multidisciplinary approach utilized in this study can be applied to investigate other proteins and facilitate the development of targeted therapies. Future exploration of the impact of point mutations on modulating the conformational space of PR65 may pave the way for the development of novel therapeutic strategies for diseases associated with PP2A dysfunction. Notably, the current integrated experimental and computational analysis helps dissect the role of individual residues in supporting the conformational mechanics of PR65. Building upon the established optical trapping approach, mmWave-THz dielectric spectroscopy emerges as a viable method for the real-time tracking of protein dipole movement and globular vibration modes. This technique offers the capability to investigate ultrafast dynamics implicated in extensive biomolecular conformational shifts on a significant scale, while maintaining a non-contact and non-intrusive nature. Thus, it presents a compelling pathway for delving into the intricacies of PR65 protein conformational alterations. By unraveling the complexities of PR65 and its role within the broader PP2A family, the study helps us move closer to unlocking the potential for targeted therapies and improved treatments for diseases linked to PP2A dysregulation.

## Methods

### System and MD simulations-

The PR65 structure resolved for the trimeric PP2A, deposited in the Protein Data Bank (PDB: 6NTS)^3^ was used as starting conformation in our simulations. PR65 is in its compact form in the trimer. The structure was simulated in TIP3 explicit water having 25 Å of water padding in all directions. Thus, providing at least 50 Å of water between PR65 and its periodic images. Ions were added to neutralize the systems and ion concentrations were set to 150 mM NACl. System size was ~ 164,147 atoms for WT PR65 simulations. All system preparations were performed in VMD and all MD simulations were performed in NAMD3^51^ using the CHARMM36 all-atom additive protein force field^52^. A time step of 2 fs was used in the simulations. Temperature was kept constant at 310 K via Langevin dynamics using a damping coefficient of 1 ps^− 1^. The pressure was kept at 1 atm using the Langevin Nosé–Hoover method with an oscillation period of 100 fs and a damping time scale of 50 fs. A cut-off distance of 12 Å was applied for van der Waals interactions. To calculate long-range electrostatic interactions, the particle-mesh Ewald method was used. PR65 mutants were generated by introducing single point mutations using the mutator plugin of VMD. PR65 mutants were simulated following the steps indicated above. Two rounds of minimization and equilibration simulations were performed prior to each production run. First, the protein was maintained in a fixed structure and the system was subjected to 10,000 energy minimization steps, which was followed by 1 ns of stabilization to equilibrate the solvent around the protein. Subsequently, a second round of minimization-equilibration was performed, where each system underwent an additional 10,000 step minimization, this time without any restrictions, which was subsequently followed by 4 ns of stabilization using harmonic constraints (of 1 kcal/mol/Å^2^) on C^α^-atoms only. Following these simulations, constraints were completely removed, and the system went through another round of 4 ns equilibration. Upon completion of this second round of minimization-equilibration, production runs were performed.

Conformations were sampled every 0.1 ns in the MD simulations and used in RMSD, RMSF, and PCA calculations. Thus, when combining the three sets of simulations for each of the WT and mutant systems, we obtained an ensemble of 19620 snapshots from a total of 3×654 ns = 1962 ns of MD simulations. Cumulatively MD simulations of 13.734 μs were evaluated. We performed all calculations using our custom analysis codes, executed in VMD and MATLAB, which also utilized some of their built-in functions.

### In silico saturation mutagenesis-

We used the Rhapsody^32, 37^ tool to carry out *in-silico* saturation mutagenesis on the apo form of PR65 (PDB: 1B3U). Rhapsody was used to predict the pathogenicity corresponding to all possible single point mutations at each residue position of PR65. We further used ProTSPoM^33^ to predict the change in Gibbs free energy of folding associated with all possible point mutations for each residue position.

### Hinge site detection-

Hinge sites within a specific ENM mode refer to regions that exhibit minimal displacements, if any, during that particular mode. Residues participating in these regions act as pivotal or anchor points, connecting substructures that move collectively around them and as such, they play a crucial mechanical role. In the GNM analysis, the hinge sites are identified as the zero-crossover points in the mode shapes generated for each mode^38, 39^. The *i*^*th*^ mode shape is obtained by plotting the elements of the *i*^*th*^ eigenvector of the *N* x *N* connectivity/Kirchhoff matrix as a function of residue index for a protein of *N* residues^38, 39^. In our study, we specifically concentrated on the global hinges found in the soft (lowest frequency) GNM modes, e.g. modes 1–6 at the lower frequency range of the mode spectrum. We used the *calcHinges* function of ProDy^53^ with the default parameters and protocol to compute the hinge sites corresponding to these global modes.

### Optical trapping experiments.

To perform the optical trapping experiment, we first make a microwell on a clean glass microscope slide of 150 μm thickness (Ted Pella, Inc.) using an imaging spacer (Secure Seal imaging spacer, Grace Bio-labs) to form an open chamber measuring 120 μm in depth and 9 mm in diameter. Using a micropipette, this chamber was filled with 10 μL of the analyte, diluted to 20 times its original concentration, and a sample of gold-on-glass, on which double nanohole (DNH) apertures have been fabricated by colloidal lithography, was inverted over it, sealing off the chamber. The sample so prepared was placed on a sample stage between a 100× oil immersion objective and a 10× collection objective, with the side of the microscope cover slide in contact with the oil immersion objective. By turning the z-knob on a 3-stage piezo-controller used to move the sample mounted on the sample stage, a focal position was reached whereupon multiple bright spots, corresponding to apertures on the gold-on-glass sample, show up on a computer screen connected to a CCD camera which captures light going through the apertures. The laser was then turned on to a very low drive current (18 mA, for example) and the x-, y- knobs of the piezo were used to move the gold-on-glass sample in a horizontal plane to select an aperture by aligning a white spot on the screen to the center of the laser’s diffraction pattern on the same screen. A half-wave plate (Thorlabs, WPH05M-980) mounted on the laser path was used to check whether the selected aperture was a DNH by rotating the half-wave plate and observing the change in signal level on a computer screen connected to a USB-4771A data acquisition module from Thorlabs. For a DNH, the change in signal level should be between 30% and 50%^28^. If an aperture failed this test, a different one was selected by the same procedure and the test was repeated until a DNH was found. The laser power was then increased to 22.5 mW before the 100× objective (corresponding to a drive current of 79 mA by our calibration), and identify trapping, observed as a discrete jump and an increase in noise of at least 10% on the screen connected to the signal acquisition module (as in Fig. 7A above). The data, acquired at a 100 kHz sampling rate, was then exported to MATLAB for analysis.

### Protein expression and purification.

Site-specific mutations were introduced to the thrombin cleavable GST-PR65-H_6_ fusion protein pRSETa plasmid using Quikchange Site-directed mutagenesis protocol (Agilent (UK) Ltd.). PR65 proteins (wild-type and mutants) were expressed in *E. coli* as described previously^54^.

### Protein stability measurements.

Protein stability was measured using a thermal shift assay on a Bio-Rad CFX Connect qPCR instrument, whereby the unfolding is detected by fluorescence of the hydrophobic dye Spyro Orange that binds to the unfolded state of the protein. The experiments were performed in clear-bottom, half-volume, 96-well plates using final well volume of 25 μL, PR65 concentration of 10 μM and Sypro Orange concentration of 0.025 μM. The samples were incubated at 25°C for 2 minutes before increasing the temperature by 0.5°C every 30 seconds up to 70°C. At each temperature, the fluorescence intensity was measured using an excitation wavelength of 471 nm and an emission wavelength of 570 nm. Data were analyzed using the GraphPad Prism software.

## Figures and Tables

**Figure 7:** This image is not available with this version.

**Table 1.** PR65 mutations distinguished by highly destabilizing and/or pathogenic effects

Location	Mutation	Repeat #, secondary structure	ΔΔG (kcal/mol)	Pathogenicity score
Interface with the regulatory subunit	M179H	Repeat 4, helix 2	1.52	0.71
	R182T	Repeat 5, helix 2	1.46	0.77
	S255F	Repeat 6, loop 1	1.47	0.86
	W256H	Repeat 6, helix 2	2.08	0.82
Interface with the catalytic subunit	W416F	Repeat 11, helix 2	1.23	0.82
	R497T	Repeat 13, helix 2	1.34	0.81

**Table 2.** Pathogenicity and conformational dynamics of the six selected mutations

Mutation	Results from theory and computations			Results from Nanoplasmonic Optical Tweezers experiments	
	Pathogenicity score [0-1]	Mean end-to-end distance (Å)	Standard deviation in end-to-end distance (Å)	Corner frequency (Hz)	Normalized RMSD
				(mean ± SD)	
S323L	0.24	70.3	9.4	122.82 ± 5.4	0.0018 ± 0.0009
F502W	0.36	70.4	9.9	45.07 ± 2.0	0.0019 ± 0.0007
WT	0	66.2	9.8	24.66 ± 2.9	0.0021 ± 0.0009
E375D	0.20	68.4	8.9	25.75 ± 5.9	0.0030 ± 0.0007
Y168V	0.18	64.6	11.2	20.97 ± 4.3	0.0033 ± 0.0006
L197V	0.04	67.2	9.7	16.78 ± 1.2	0.0067 ± 0.0015
D315E	0.03	58.2	15.6	15.99 ± 3.1	0.0099 ± 0.0028

## Data Availability

All data supporting the findings of this study are available within the paper and its Supplementary Information. All source data will be provided on request.

## References

[1] Hunter T. Protein kinases and phosphatases: the yin and yang of protein phosphorylation and signaling. Cell 80, 225–236 (1995). 7834742 10.1016/0092-8674(95)90405-0

[2] Sangodkar J. All roads lead to PP 2A: exploiting the therapeutic potential of this phosphatase. The FEBS journal 283, 1004–1024 (2016). 26507691 10.1111/febs.13573 PMC4803620

[3] Leonard D. Selective PP2A Enhancement through Biased Heterotrimer Stabilization. Cell 181, 688–701.e616 (2020). 32315618 10.1016/j.cell.2020.03.038 PMC7243596

[4] Allen-Petersen B.L. Activation of PP2A and inhibition of mTOR synergistically reduce MYC signaling and decrease tumor growth in pancreatic ductal adenocarcinoma. Cancer research 79, 209–219 (2019). 30389701 10.1158/0008-5472.CAN-18-0717 PMC6318036

[5] Tohmé R. Direct activation of PP2A for the treatment of tyrosine kinase inhibitor–resistant lung adenocarcinoma. JCI insight 4 (2019). 10.1172/jci.insight.125693 PMC6478418 30830869

[6] Kauko O. PP2A inhibition is a druggable MEK inhibitor resistance mechanism in KRAS-mutant lung cancer cells. Science translational medicine 10, eaaq1093 (2018). 10.1126/scitranslmed.aaq1093 PMC8335581 30021885

[7] Cristóbal I. Deregulation of the PP2A Inhibitor SET Shows Promising Therapeutic Implications and Determines Poor Clinical Outcome in Patients with Metastatic Colorectal CancerSignificance of SET Overexpression in Colorectal Cancer. Clinical cancer research 21, 347–356 (2015). 25388166 10.1158/1078-0432.CCR-14-0724

[8] Gutierrez A. Phenothiazines induce PP2A-mediated apoptosis in T cell acute lymphoblastic leukemia. J Clin Inv 124, 644–655 (2014). 10.1172/JCI65093 PMC3904599 24401270

[9] Rincón R. PP2A inhibition determines poor outcome and doxorubicin resistance in early breast cancer and its activation shows promising therapeutic effects. Oncotarget 6, 4299 (2015). 25726524 10.18632/oncotarget.3012 PMC4414191

[10] Brisson M. Discovery and characterization of novel small molecule inhibitors of human Cdc25B dual specificity phosphatase. Mol Pharmacol 66, 824–833 (2004). 15231869 10.1124/mol.104.001784

[11] Lui V.W. Frequent mutation of receptor protein tyrosine phosphatases provides a mechanism for STAT3 hyperactivation in head and neck cancer. Proc Natl Acad Sci USA 111, 1114–1119 (2014). 24395800 10.1073/pnas.1319551111 PMC3903220

[12] Brautigan D.L. & Shenolikar S. Protein serine/threonine phosphatases: keys to unlocking regulators and substrates. Ann Rev Biochem 87, 921–964 (2018). 29925267 10.1146/annurev-biochem-062917-012332

[13] Junttila M.R. CIP2A inhibits PP2A in human malignancies. Cell 130, 51–62 (2007). 17632056 10.1016/j.cell.2007.04.044

[14] Tsujio I. Inhibitors of protein phosphatase-2A from human brain structures, immunocytological localization and activities towards dephosphorylation of the Alzheimer type hyperphosphorylated tau. FEBS letters 579, 363–372 (2005). 15642345 10.1016/j.febslet.2004.11.097

[15] O’Connor C.M., Perl A., Leonard D., Sangodkar J. & Narla G. Therapeutic targeting of PP2A. The international journal of biochemistry & cell biology 96, 182–193 (2018). 29107183 10.1016/j.biocel.2017.10.008 PMC5927617

[16] Kaynak B.T. Cooperative mechanics of PR65 scaffold underlies the allosteric regulation of the phosphatase PP2A. Structure 31, 607–618 (2023). 36948205 10.1016/j.str.2023.02.012 PMC10164121

[17] Haesen D. Recurrent PPP2R1A Mutations in Uterine Cancer Act through a Dominant-Negative Mechanism to Promote Malignant Cell GrowthMechanism of Action of PP2A Aα Subunit Cancer Mutants. Cancer research 76, 5719–5731 (2016). 27485451 10.1158/0008-5472.CAN-15-3342

[18] O’Connor C.M. Inactivation of PP2A by a recurrent mutation drives resistance to MEK inhibitors. Oncogene 39, 703–717 (2020). 31541192 10.1038/s41388-019-1012-2 PMC6980487

[19] Ruediger R., Ruiz J. & Walter G. Human cancer-associated mutations in the Aα subunit of protein phosphatase 2A increase lung cancer incidence in Aα knock-in and knockout mice. Molecular and cellular biology 31, 3832–3844 (2011). 21791616 10.1128/MCB.05744-11 PMC3165721

[20] Tsytlonok M. Complex energy landscape of a giant repeat protein. Structure 21, 1954–1965 (2013). 24120762 10.1016/j.str.2013.08.028 PMC4256716

[21] Lee G. Nanospring behaviour of ankyrin repeats. Nature 440, 246–249 (2006). 16415852 10.1038/nature04437

[22] Chen W., Lu W., Wolynes P.G. & Komives E.A. Single-molecule conformational dynamics of a transcription factor reveals a continuum of binding modes controlling association and dissociation. Nucleic Acids Res 49, 11211–11223 (2021). 34614173 10.1093/nar/gkab874 PMC8565325

[23] Halder K. MD simulations and FRET reveal an environment-sensitive conformational plasticity of importin-β. Biophys J 109, 277–286 (2015). 26200863 10.1016/j.bpj.2015.06.014 PMC4621615

[24] Cho U.S. & Xu W. Crystal structure of a protein phosphatase 2A heterotrimeric holoenzyme. Nature 445, 53–57 (2007). 17086192 10.1038/nature05351

[25] Sabath K. & Jonas S. Take a break: Transcription regulation and RNA processing by the Integrator complex. Curr Opin Struct Biol 77, 102443 (2022). 36088798 10.1016/j.sbi.2022.102443

[26] Yang L.W. & Bahar I. Coupling between catalytic site and collective dynamics: a requirement for mechanochemical activity of enzymes. Structure 13, 893–904 (2005). 15939021 10.1016/j.str.2005.03.015 PMC1489920

[27] Atilgan A.R. Anisotropy of fluctuation dynamics of proteins with an elastic network model. Biophys J 80, 505–515 (2001). 11159421 10.1016/S0006-3495(01)76033-X PMC1301252

[28] Hajisalem G. Accessible high-performance double nanohole tweezers. Opt Express 30, 3760–3769 (2022). 35209628 10.1364/OE.446756

[29] Liang F., Guo Y., Hou S. & Quan Q. Photonic-plasmonic hybrid single-molecule nanosensor measures the effect of fluorescent labels on DNA-protein dynamics. Sci Adv 3, e1602991 (2017). 28560341 10.1126/sciadv.1602991 PMC5446212

[30] Pang Y. & Gordon R. Optical trapping of a single protein. Nano Lett 12, 402–406 (2012). 22171921 10.1021/nl203719v

[31] Peri S.S.S. Quantification of low affinity binding interactions between natural killer cell inhibitory receptors and targeting ligands with a self-induced back-action actuated nanopore electrophoresis (SANE) sensor. Nanotechnology 32, 045501 (2021). 33027774 10.1088/1361-6528/abbf26 PMC8346883

[32] Ponzoni L., Peñaherrera D.A., Oltvai Z.N. & Bahar I. Rhapsody: predicting the pathogenicity of human missense variants. Bioinformatics 36, 3084–3092 (2020). 32101277 10.1093/bioinformatics/btaa127 PMC7214033

[33] Banerjee A. & Mitra P. Estimating the Effect of Single-Point Mutations on Protein Thermodynamic Stability and Analyzing the Mutation Landscape of the p53 Protein. J Chem Inf Model 60, 3315–3323 (2020). 32401507 10.1021/acs.jcim.0c00256

[34] Booth L.S. Modelling of the dynamic polarizability of macromolecules for single-molecule optical biosensing. Sci Rep 12, 1995 (2022). 35132077 10.1038/s41598-022-05586-0 PMC8821610

[35] Groves M.R., Hanlon N., Turowski P., Hemmings B.A. & Barford D. The structure of the protein phosphatase 2A PR65/A subunit reveals the conformation of its 15 tandemly repeated HEAT motifs. Cell 96, 99–110 (1999). 9989501 10.1016/s0092-8674(00)80963-0

[36] Grinthal A., Adamovic I., Weiner B., Karplus M. & Kleckner N. PR65, the HEAT-repeat scaffold of phosphatase PP2A, is an elastic connector that links force and catalysis. Proc Natl Acad Sci USA 107, 2467–2472 (2010). 20133745 10.1073/pnas.0914073107 PMC2823866

[37] Ponzoni L. & Bahar I. Structural dynamics is a determinant of the functional significance of missense variants. Proc Natl Acad Sci USA 115, 4164–4169 (2018). 29610305 10.1073/pnas.1715896115 PMC5910821

[38] Bahar I., Atilgan A.R. & Erman B. Direct evaluation of thermal fluctuations in proteins using a single-parameter harmonic potential. Fold Des 2, 173–181 (1997). 9218955 10.1016/S1359-0278(97)00024-2

[39] Haliloglu T., Bahar I. & Erman B. Gaussian dynamics of folded proteins. Phys Rev Lett 79, 3090 (1997).

[40] Gur M. Molecular dynamics simulations of site point mutations in the TPR domain of cyclophilin 40 identify conformational states with distinct dynamic and enzymatic properties. J Chem Phys 148, 145101 (2018). 29655319 10.1063/1.5019457 PMC5891347

[41] Gur M., Cheng M.H., Zomot E. & Bahar I. Effect of Dimerization on the Dynamics of Neurotransmitter:Sodium Symporters. J Phys Chem B 121, 3657–3666 (2017). 28118712 10.1021/acs.jpcb.6b09876 PMC5402697

[42] Bustamante C.J., Chemla Y.R., Liu S. & Wang M.D. Optical tweezers in single-molecule biophysics. Nat Rev Methods Primers 1 (2021). 10.1038/s43586-021-00021-6 PMC8629167 34849486

[43] Svoboda K. & Block S.M. Biological applications of optical forces. Annu Rev Biophys Biomol Struct 23, 247–285 (1994). 7919782 10.1146/annurev.bb.23.060194.001335

[44] Naqvi M.M. Protein chain collapse modulation and folding stimulation by GroEL-ES. Sci Adv 8, eabl6293 (2022). 35245117 10.1126/sciadv.abl6293 PMC8896798

[45] Raza M.U., Peri S.S.S., Ma L.C., Iqbal S.M. & Alexandrakis G. Self-induced back action actuated nanopore electrophoresis (SANE). Nanotechnology 29, 435501 (2018). 30073973 10.1088/1361-6528/aad7d1

[46] Yang W., van Dijk M., Primavera C. & Dekker C. FIB-milled plasmonic nanoapertures allow for long trapping times of individual proteins. iScience 24, 103237 (2021). 34746702 10.1016/j.isci.2021.103237 PMC8551080

[47] Yoo D. Low-Power Optical Trapping of Nanoparticles and Proteins with Resonant Coaxial Nanoaperture Using 10 nm Gap. Nano Lett 18, 3637–3642 (2018). 29763566 10.1021/acs.nanolett.8b00732

[48] Yousefi A. Optical Monitoring of In Situ Iron Loading into Single, Native Ferritin Proteins. Nano Lett 23, 3251–3258 (2023). 37053043 10.1021/acs.nanolett.3c00042 PMC10141409

[49] Ying C. Watching single unmodified enzymes at work. arXiv preprint arXiv:2107.06407 (2021).

[50] Anyika T., Hong C. & Ndukaife J.C. High-speed nanoscale optical trapping with plasmonic double nanohole aperture. Nanoscale 15, 9710–9717 (2023). 37132641 10.1039/d2nr07073a

[51] Phillips J.C. Scalable molecular dynamics on CPU and GPU architectures with NAMD. J Chem Phys 153, 044130 (2020). 32752662 10.1063/5.0014475 PMC7395834

[52] Best R.B. Optimization of the additive CHARMM all-atom protein force field targeting improved sampling of the backbone ϕ, ψ and side-chain χ1 and χ2 dihedral angles. J Chem Theory Comput 8, 3257–3273 (2012). 23341755 10.1021/ct300400x PMC3549273

[53] Krieger J.M., Sorzano C.O.S., Carazo J.M. & Bahar I. Protein dynamics developments for the large scale and cryoEM: case study of ProDy 2.0. Acta Crystallogr D Struct Biol 78, 399–409 (2022). 35362464 10.1107/S2059798322001966 PMC8972803

[54] Synakewicz M., Bauer D., Rief M. & Itzhaki L.S. Bioorthogonal protein-DNA conjugation methods for force spectroscopy. Sci Rep 9, 13820 (2019). 31554828 10.1038/s41598-019-49843-1 PMC6761116

